# Supplementary material for: Measuring the influence of depressive symptoms on engagement, adherence, and weight loss in an eHealth intervention
Source: PLOS Digit Health. 2025 Mar 25;4(3):e0000766. doi: 10.1371/journal.pdig.0000766 (PMC11936179; doi:10.1371/journal.pdig.0000766)

PLOS Digital Health

Measuring the influence of depressive symptoms on engagement, adherence, and weight loss in a 6-month eHealth intervention

--Manuscript Draft--

Manuscript Number:	PDIG-D-24-00324
Article Type:	Research Article
Full Title:	Measuring the influence of depressive symptoms on engagement, adherence, and weight loss in a 6-month eHealth intervention
Short Title:	Depressive symptom association with engagement, adherence, and weight loss
Corresponding Author:	Lex Hurley, Ph.D., M.P.H<br>UNC-Chapel Hill: The University of North Carolina at Chapel Hill<br>Chapel Hill, NC UNITED STATES OF AMERICA
Order of Authors:	Lex Hurley, Ph.D., M.P.H
	Nisha G. O'Shea, Ph.D.
	Julianne Power, Ph.D.
	Christopher Sciamanna, M.D.
	Deborah F. Tate, Ph.D.
Keywords:	eHealth; mHealth; engagement; adherence; depression; weight; obesity; website
Abstract:	Background: Digital behavior change interventions (eHealth, mHealth) are known to be capable of promoting clinically significant weight loss among some participants. However, these programs can struggle with declining engagement and adherence over time, which can hamper their effectiveness. This analysis examines the extent that depression symptoms may negatively influence engagement, adherence, and 6 month weight change in an eHealth intervention. Methods: Two structural equation models are tested which map the effects of baseline depression symptoms on weight change outcomes, mediated through latent constructs of engagement and adherence, respectively. Results: Depression symptoms showed no direct association with weight change, but were negatively associated with both constructs of engagement and adherence, which in turn were negatively associated with weight change in both models. It was determined depression symptoms had a positive indirect association with weight change fully mediated through these variables, meaning less weight loss or possible weight gain. Discussion: This analysis shows that depression symptoms had a significant, undesirable effect on weight loss outcomes within this eHealth intervention, fully mediated through measured participant engagement and adherence. Further research is needed to identify means to address this issue in future digital interventions.
Additional Information:	
**Question**	**Response**
**Government Employee**<br><br>Are you or any of the contributing authors an employee of the United States government?<br><br>Manuscripts authored by one or more US Government employees are not copyrighted, but are licensed under a CC0 Public Domain Dedication, which allows	No - No authors are employees of the U.S. government.

Powered by Editorial Manager® and ProduXion Manager® from Aries Systems Corporation

unlimited distribution and reuse of the article for any lawful purpose. This is a legal requirement for US Government employees.<br><br>This will be typeset if the manuscript is accepted for publication.	
**Financial Disclosure**<br><br>Enter a financial disclosure statement that describes the sources of funding for the work included in this submission. Review the submission guidelines for detailed requirements. View published research articles from *PLOS Digital Health* for specific examples.<br><br>This statement is required for submission and **will appear in the published article** if the submission is accepted. Please make sure it is accurate.<br><br>**Funded studies**<br>Enter a statement with the following details:<br>• Initials of the authors who received each award<br>• Grant numbers awarded to each author<br>• The full name of each funder<br>• URL of each funder website<br>• Did the sponsors or funders play any role in the study design, data collection and analysis, decision to publish, or preparation of the manuscript?<br><br>Did you receive funding for this work?	Yes
Please add funding details.<br>as follow-up to "**Financial Disclosure**<br><br>Enter a financial disclosure statement that describes the sources of funding for the work included in this submission. Review the submission guidelines for detailed requirements. View published research articles from *PLOS Digital Health* for specific examples.	This research was supported by a grant from the T32 Cancer Health Disparities Training Grant from the National Cancer Institute of the National Institutes of Health (T32CA128582).

This statement is required for submission and **will appear in the published article** if the submission is accepted. Please make sure it is accurate.<br><br>**Funded studies**<br>Enter a statement with the following details:<br>• Initials of the authors who received each award<br>• Grant numbers awarded to each author<br>• The full name of each funder<br>• URL of each funder website<br>• Did the sponsors or funders play any role in the study design, data collection and analysis, decision to publish, or preparation of the manuscript?<br><br>Did you receive funding for this work?"	
Please select the country of your main research funder (please select carefully as in some cases this is used in fee calculation).<br>as follow-up to "**Financial Disclosure**<br><br>Enter a financial disclosure statement that describes the sources of funding for the work included in this submission. Review the submission guidelines for detailed requirements. View published research articles from *PLOS Digital Health* for specific examples.<br><br>This statement is required for submission and **will appear in the published article** if the submission is accepted. Please make sure it is accurate.<br><br>**Funded studies**<br>Enter a statement with the following details:<br>• Initials of the authors who received each award<br>• Grant numbers awarded to each author<br>• The full name of each funder<br>• URL of each funder website<br>• Did the sponsors or funders play any role in the study design, data collection and analysis, decision to publish, or preparation of the manuscript?	UNITED STATES - US

Did you receive funding for this work?"	
**Competing Interests**<br><br>On behalf of all authors, disclose any competing interests that could be perceived to bias this work.<br><br>This statement will be typeset if the manuscript is accepted for publication.<br><br>Review the instructions link below and PLOS Digital Health's competing interests policy to determine what information must be disclosed at submission.	The authors have no conflicts of interest to disclose.
**Data Availability**<br><br>Provide a **Data Availability Statement** in the box below. This statement should detail where the data used in this submission can be accessed. This statement will be typeset if the manuscript is accepted for publication.<br><br>Before publication, authors are required to make all data underlying their findings fully available, without restriction. Review our PLOS Data Policy page for detailed information on this policy. Instructions for writing your Data Availability statement can be accessed via the Instructions link below.	The authors do not have permission to share raw study data due to requirements to protect the privacy of participants, in accordance with their informed consent. However, de-identified data used in this specific analysis and/or output files related to this analysis will be made available upon request to the corresponding author.

Powered by Editorial Manager® and ProduXion Manager® from Aries Systems Corporation

Manuscript

Click here to access/download;Manuscript;LNPA_SEM_Manuscript_PLoS_DH

Full Title:

Measuring the influence of depressive symptoms on engagement, adherence, and weight loss in a 6-month eHealth intervention

Short Title:

Depressive symptom association with engagement, adherence, and weight loss

Lex Hurley[1]*, Nisha G. O'Shea[2], Julianne Power[1], Christopher Sciamanna[3], Deborah F. Tate[1,4,5]

[1] The University of North Carolina at Chapel Hill, Department of Health Behavior, Chapel Hill, North Carolina, United States

[2] Research Triangle Institute (RTI) International, Research Triangle Park, North Carolina, United States

[3] Penn State Cancer Institute, Department of Medicine, Hershey, Pennsylvania, United States

[4] The University of North Carolina at Chapel Hill, Department of Nutrition, Chapel Hill, North Carolina, United States

[5] Nutrition Research Institute, University of North Carolina at Chapel Hill, Kannapolis, NC, United States

* Corresponding author

E-mail: lexh@unc.edu (LH)

ORCID: https://orcid.org/0000-0003-2217-0492

Abstract

Background: Digital behavior change interventions (eHealth, mHealth) are known to be capable of promoting clinically significant weight loss among some participants. However, these programs can struggle with declining engagement and adherence over time, which can hamper their effectiveness. This analysis examines the extent that depression symptoms may negatively influence engagement, adherence, and 6 month weight change in an eHealth intervention. **Methods**: Two structural equation models are tested which map the effects of baseline depression symptoms on weight change outcomes, mediated through latent constructs of engagement and adherence, respectively. **Results**: Depression symptoms showed no direct association with weight change, but were negatively associated with both constructs of engagement and adherence, which in turn were negatively associated with weight change in both models. It was determined depression symptoms had a positive indirect association with weight change fully mediated through these variables, meaning less weight loss or possible weight gain. **Discussion**: This analysis shows that depression symptoms had a significant, undesirable effect on weight loss outcomes within this eHealth intervention, fully mediated through measured participant engagement and adherence. Further research is needed to identify means to address this issue in future digital interventions.

Author Summary

Digital behavior change interventions (DBCIs) have shown promise for developing scalable interventions to help address the growing obesity epidemic. However, it is known that engagement with DBCIs tends to drop off before an intervention is finished, thus reducing its

potential for effectiveness. Depression is often mentioned as a likely contributor to reduced engagement and/or adherence in these programs, but is rarely studied in-depth outside of mental health-specific interventions. We applied structural equation modeling to test the extent that depression may influence weight change outcomes directly, or mediated through latent constructs of engagement and adherence to try and solidify the literature. We found that depression symptoms were fully mediated through these constructs; meaning that higher levels of depression had negative impacts on engagement and adherence, which in turn have negative impacts on weight (i.e., more weight loss). We concluded that depression symptoms have a significant indirect positive effect on weight, meaning less weight loss or possible weight gain. Our study provides a basis to consider baseline depression levels as an important variable to consider when designing future DBCIs for weight management, and possibly other outcomes.

Introduction

Digital behavior change interventions (DBCIs) are able to benefit a variety of health outcomes, including weight management, physical activity promotion, as well as smoking and other substance use cessation; often eliciting equal or greater effect sizes than traditional in-person interventions.(1-5) These programs feature increased scalability to reach multitudes of participants for lower cost than in-person programs and are largely accessible at participants' convenience. As over 70% of U.S. adults are living with overweight or obesity, which increase risks of numerous chronic diseases, certain types of cancers, and other morbidities, this improved scalability is a key asset to support contemporary weight management interventions.(6, 7) While the literature is robust with recommendations and resources to design and implement DBCIs for a variety of health outcomes, their effectiveness inevitably depends on how much participants

use these programs. This analysis investigates one potential issue which may be contributing to reduced participant engagement, adherence, and success in DBCIs.

Participant engagement and adherence are recognized as two necessary, but not sufficient, constructs for DBCI effectiveness which happen more or less in tandem during an intervention. Despite their importance to DBCI research, definitions of these constructs have tended to vary between publications.(8) Definitions and operationalizations are provided here to promote clarity and comparability.

Engagement is defined here as the extent of meaningful usage and interaction with the digital interface of an intervention, based on the systematic definition proposed by Perski et al.(8) This latent construct may be measured using objective usage indicators including logins, page views, proportion of full program components utilized, as well as messages, resources or lessons read.(9, 10) Engagement with DBCIs is typically considered essential to promote the dose received of intervention content and is a common focus of DBCI research. There is an often-cited 'Law of Attrition' which broadly states that once the perceived novelty or usefulness of a digital program has faded, there is a rapid decline in user engagement, an increased risk of disengagement (i.e., all usage indicators dropping to zero, but not lost to follow-up; a.k.a. "non-usage attrition"), and possible attrition or loss to follow-up.(11-15) Because of this, much DBCI research focuses on increasing or sustaining program engagement among active participants; though comparatively less research investigates factors which may negatively influence participant engagement and adherence.

Adherence is defined here as the degree that a participant's actual (measured) behavior corresponds with requests or goals from an intervention, based on definitions of adherence from the WHO,(16-18) and is generally similar to definitions used in other contemporary

publications.(19-21) In weight management programs, these recommendations would typically include adherence to dietary regimens,(22) physical activity goals(23), daily weighing recommendations, attendance to counseling sessions, and regular self-monitoring of behaviors and outcomes;(24, 25) to contribute to improved weight outcomes.(26-28)

Depression and poor mental health have been anecdotally acknowledged as probable contributors to reduced engagement, adherence, and overall performance in DBCIs for weight management and other health outcomes.(8, 20, 26) However, the relationships are rarely quantified in digital interventions outside of studies specifically focusing on mental health outcomes. In the few cases when depression symptoms are directly assessed in weight management interventions, they rarely predict significant variance in weight change.(29-31) One possible explanation might be regression to the mean, as commonly applied measures for depression are positively correlated with body weight, such that higher bodyweights are associated with higher depressive symptoms and vice versa (not considering very low bodyweights), and reductions in bodyweight may improve measured depression symptoms.(29, 32, 33) Another possibility may be that depression symptoms exacerbate the risk of participant disengagement and/or dropout in studies, and their influences are not accurately considered in analyses due to missing data.(8)

In their systematic review, Perski *et al* identify several studies which mention isolated associations between mental health and differential adherence or engagement in DBCIs, and possibly signaling increased risk of participant dropout.(8, 18, 34-43) Wing, Phelan, and Tate found that baseline depression symptoms negatively influenced mediating program adherence, and thus weight change within an in-person intervention.(26) Additionally in their systematic review of factors influencing adherence in DBCIs for noncommunicable diseases, Jakob et al

found that depression symptoms were reported to be negatively associated with adherence for several outcomes including physical activity and weight loss, and only positively associated with adherence in DBCIs specifically targeting depression.(20) This analysis seeks to test how depression symptoms may influence distal weight change outcomes when modeled through engagement and adherence as mediators.

The guiding hypotheses for this analysis are: 1) depression symptoms will have a negative influence on the latent constructs of engagement and adherence; 2) engagement and adherence will each be negatively associated with 6-month weight change (i.e. weight loss); and 3) the direct effect of depressive symptoms on weight change will be fully mediated by program engagement and adherence.

Materials and Methods

Dataset

Data for this analysis come from LoseNow PA (LNPA), an NIH-funded 12-month cluster-randomized controlled eHealth DBCI for weight management among primary care patients living with overweight or obesity (clinicaltrials.gov identifier: NCT01606813).(44) In this study, primary care providers (PCPs; k = 31) were randomly assigned to one of three intervention groups, and patients (N = 550) received the intervention assigned to their provider: 1) enhanced usual care (EUC; n = 187); 2) internet weight loss intervention (IWL; n = 181); or 3) internet weight loss intervention plus automated physician-tailored advice (IWL+PCP; n = 182).(44) Summarizing primary outcomes of the trial, both the IWL and IWL+PCP arms exhibited significantly greater weight loss than EUC, and there were no significant differences between

IWL and IWL+PCP on any outcomes. Full details of the trial can be found in Tate *et al.*, 2022.(44)

Both IWL intervention arms had access to a study website requiring a username and password to log in. The study website included instructional lessons and resources; self-monitoring diary pages for diet, physical activity, and weight; weekly computer-tailored feedback messages and graphs; personal goal setting and problem-solving tools; progress summary pages; a social forum to message with other participants; as well as opt-in reminder text messages containing updates, encouragement, and motivational content. Participants could choose from several Eating and Activity Plan monitoring options to customize how they monitored calories and how exercise goals would progress, respectively, and shift their monitoring plan(s) if desired during the intervention. The primary difference between the intervention arms was that participants in the IWL+PCP condition also received brief biweekly emails containing a computer-generated tailored message addressed from their PCP related to weight loss progress, frequency of website log-ins, time in the program, and other aspects such as patient-reported motivation. PCP’s could view and edit these messages for each patient before sending, though results showed that this functionality was rarely used by PCPs (only 1.2% of all PCP messages sent were edited).(44)

Since the study website was functionally identical across both intervention arms and no significant influence of the automated physician advice messaging was found, participants from both arms were pooled to maximize the statistical power of this secondary analysis. Of the full N = 363 participants assigned to the IWL and IWL+PCP groups, 24 did not ever log in to the website and are excluded from this analysis, bringing the effective sample size to n = 339. Additionally, this analysis specifically focuses on the first 6 program months to best measure the

effects of active program engagement and adherence with the following rationale: This timeframe is critical as most DBCI engagement occurs in the early months of an intervention, most weight loss occurs during months 1-6, and users are increasingly likely to disengage from DBCIs as duration increases in months 6-12.(12, 45-47)

Ethics Statement

The parent LNPA study was approved by institutional review boards at the University of North Carolina at Chapel Hill (#12-1661) and Penn State College of Medicine (#39237). Both PCPs and patients completed written informed consent forms prior to data collection and randomization, as described in Tate *et al*, 2022. (44)

Measures

LNPA participants completed study assessments at baseline, 3, 6, and 12 months. During baseline assessments, participants self-reported various sociodemographic characteristics including age, sex, and race/ethnicity, which are applied as exogenous covariates. Weight change is measured by a difference score between participant weights at baseline and 6 months, and is the primary dependent variable of analysis. Sensitivity analyses using 12-month weight change data, as well as percent weight change at 6 and 12 months, were applied for added confidence in estimates.

The Centers for Epidemiology – Depression (CES-D) scale was administered as part of baseline study assessments and is a validated measure of depression symptoms.(48) Briefly, the CES-D is a non-diagnostic psychometric scale with score values ranging from 0-60, with higher values indicating stronger depression symptoms, and scores ≥16 commonly referenced as

indicating clinical (severe) depression.(48) Standardized CES-D scores were used as the primary exogenous predictor of interest in the following simultaneous equation models.

Indicators of engagement with the IWL study website include logins, page hits, personal goals created, and social forum posts – all of which have been previously validated as indicators for this construct.(9) Website page hits refer to the number of time-stamped website URLs visited by logged user IDs. To reduce collinearity with other indicators, the page hits variable only encompasses URLs that did not include the login page, home page, any of the behavioral self-monitoring pages, or the contact/help page. Therefore, increasing values of this variable indicate users accessing more lessons, resources, viewing feedback pages, etc. for a greater breadth of program usage. On the first website login each week, participants could create and track up to 3 personal goals, which were summed as an additional engagement indicator after being found to be associated with weight loss in a previous analysis.(49) The social forum posts variable is a sum of the number of posts users made on the IWL message boards where participants could communicate with other participants and LNPA staff if desired.

Indicators of adherence with LNPA program recommendations include the number of weeks the minimum physical activity goals were met, days of complete dietary logging, as well as days bodyweight was logged, and were operationalized as follows: The number of weeks that users logged a number of active minutes of exercise that met or exceeded the lowest intensity Activity Plan goals for that point in time were summed to indicate meeting minimum program goals for that week. The number of days participants either directly logged or adjusted dietary entries to include $\geq$ 800 calories were summed to indicate complete days of dietary monitoring, based on previous literature which indicated that this much logging is associated with improved weight management.(50, 51) Lastly, days that users self-monitored their bodyweight were

summed to demonstrate additional adherence to program recommendations, as the website had dedicated pages and feedback graphs for tracking weight change over time.

Data Analysis

Missing Data

At 6 months, 26.7% of participants were missing weight measurements, and 17.6% were missing 12-month weight measurements. No clear patterns of missingness were discernible. Assuming these observations are missing at random, we applied multiple imputation using chained equations to pool point and standard error estimates across m = 50 iterations, including the most recent participant weight logged on the IWL website before 6 months in the dataset to facilitate weight imputations.(52-58)

Structural Equation Modeling

We used a structural equation modeling (SEM) approach to efficiently model mediation pathways between engagement and adherence as latent variables (LVs), and applied log-link transformations of indicator variables where appropriate. LVs of engagement and adherence were separately examined using confirmatory factor analysis to determine optimal loading variables: sum logins for engagement and sum weeks PA goals met for adherence (indicated by the "*" symbols in Fig 1). We first conducted measurement models of the adherence and engagement LVs, comparing the fit of a 1- and 2- factor model. During this phase, we found that the two LVs were highly correlated with one-another (>95%) when entered into the same model. Because of this, it was deemed necessary to estimate two separate mediation models to test study hypotheses. Path diagrams of these twin models are displayed in Fig 1.

Fig 1. Structural equation model path diagrams

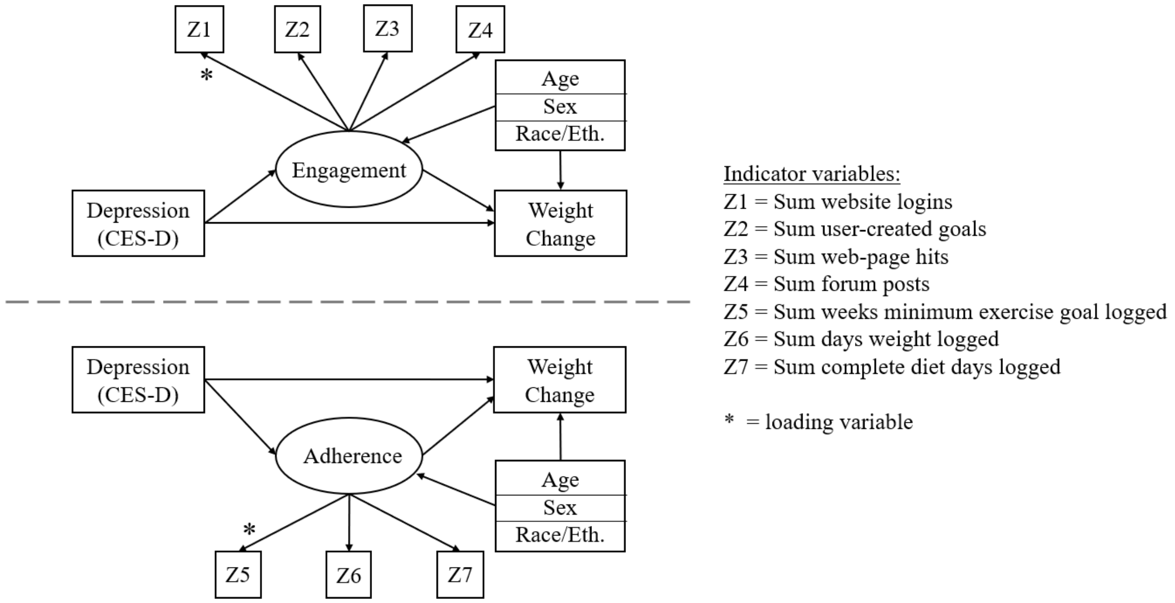

Describing the paths of these SEMs, each model estimates the direct effects of exogenous baseline depression symptoms measured by the CES-D on 6-month weight change, as well as on endogenous LVs of engagement and adherence, which in turn each have a direct effect mapped onto 6-month weight change. Three separate exogenous variables of participant age, sex, and race/ethnicity are mapped onto adherence, engagement, and weight change as demographic control covariates.

Model fit was assessed using Chi-square (χ^2) fit test, root mean square error of approximation (RMSEA), confirmatory factor index (CFI), and Tucker Lewis index (TLI) values, as well as standardized root mean square residuals (SRMR). All analyses were conducted using the *lavaan*, *semTools*, and *mice* packages for R statistical software.(58, 59)

Results

Descriptive statistics

Most LNPA participants randomized to intervention arms (N = 363) were white (82.3%) and female (70.3%) with a mean baseline weight of 97.99 kg (216.03 lbs) and average age 51.86 years. Over half of the sample (~56.7%) reported having less than a Bachelor's degree or equivalent education. Average CES-D scores at baseline were 13.13 and ranged between 0-41, with 112 (30.9%) participants reporting scores above the threshold of ≥16 indicating possible clinical depression. A full descriptive summary of baseline characteristics for those with data is displayed in Table 1. Two participants were identified as outliers with excessive weight losses in this sample; however, their inclusion did not meaningfully influence model results, so they remained in this analysis. There were no significant differences in any variables between the full and subset samples.

Table 1. Participant characteristics of pooled intervention groups (N = 363)

Variable	Frequency (%)	Mean (SD)	Min; Max
**Gender**			
Female	255 (70.3%)		
Male	93 (25.6%)		
**Race**			
White	299 (82.3%)		
Black	45 (12.5%)		
Other POC	19 (5.2%)		
**Highest Education Achieved**			
High School	89 (24.5%)		
1-3 Years College	117 (32.2%)		
4+ Years College	139 (38.3%)		
**Age**		51.86 (10.86)	21; 70
**Clinic Weight, Baseline** (kg)		97.99 (18.73)	62.1; 148.6
**Clinic Weight, 6 months** (kg)		92.63 (18.23)	57.6; 157.75
**Clinic Weight, 12 months** (kg)		93.61 (18.72)	58.2; 167.60
**6-month Weight Change** (kg)		-4.72 (5.79)	-30.0; +6.9
**CES-D Scores** (0-60)		13.13 (9.96)	0; 41

Notes:
SD = Standard Deviation; POC = People of Color; CES-D = Centers for Epidemiology Scale – Depression

Website engagement indicators exhibited high variability. Approximately 53 participants logged in 5 times or fewer over 6 months, while there were several users with extremely high engagement indicators (e.g., one user had 977 website logins and 4,446 registered page views within 6 months, compared to the sample average of approximately 99 logins and 300 page views). This high variance in engagement indicators persisted even after outlier testing and removal. Adherence indicators exhibited much lower variability by comparison. Descriptive statistics for all indicator variables are summarized in Table 2.

Table 2. LNPA engagement and adherence indicator descriptive statistics (n = 339)

Indicator Variables	Mean (SD)	Median	Min; Max
**Engagement**			
Sum logins	106.04 (182.59)	39	1; 1362
Sum page-hits	321.15 (559.69)	114	0; 4714
Sum goals set	25.05 (23.72)	18	0; 81
Sum forum posts	1.81 (4.98)	0	0; 36
**Adherence**			
Sum weeks physical activity goal met	9.85 (10.29)	5	0; 27
Sum complete diet days logged	55.59 (63.20)	27	0; 182
Sum weight days logged	91.51 (110.64)	38	0; 379

Notes:
SD = Standard Deviation

Simultaneous Equation Model Fit and Estimates

Both models converged across m = 50 imputed datasets with good fit statistics overall. The chi square (χ^2) test statistics for engagement were both significant (p's $\leq$ 0.001) which is undesirable, yet not surprising as the χ^2 test has strict assumptions which are easily violated and

less reliable among small samples.(52) Pooled RMSEAs for engagement = 0.063 (p = 0.19) and adherence = 0.089 (p = 0.02) indicate that both models fits the data fairly well, though the engagement model shows better fit. Pooled SRMRs for engagement = 0.033 and adherence = 0.023 which both indicate good fit. Additionally, the pooled CFIs for engagement = 0.971 and adherence = 0.974, as well as the pooled TLIs for engagement = 0.949 and adherence = 0.943, which all indicate good model fit for the data. and SRMR = 0.022, which all indicate good model fit for the data. These results are re-summarized in Table 3.

Table 3. SEM Fit Statistic Summary

**Indicator**	**Value (p-value)**	
	**Engagement**	**Adherence**
Chi Square (χ^2)	39.58 (p = 0.001)	37.066 (p < 0.001)
RMSEA	0.063 (p = 0.189)	0.089 (p = 0.016)
CFI	0.971	0.974
TLI	0.949	0.943
SRMR	0.033	0.023

There were no observed direct effects between baseline depression symptoms and 6-month weight change in either of the engagement and adherence models (p's ≥ 0.6). However, results indicate that a one standard deviation increase in baseline CES-D scores was negatively associated with engagement ($\gamma_{eng,dep}$ = -0.315; SE = 0.082; p < 0.001) and with adherence ($\gamma_{adh,dep}$ = -0.333; SE = 0.083; p < 0.001), net of demographic covariates. In turn, these constructs were each associated with significant reductions in weight at 6 months, net of demographic covariates. As program engagement increased, participants lost an additional 2.1 kg by 6-months ($\gamma_{wtch,eng}$ = -2.097; SE = 0.209; p < 0.001), controlling for covariates. Further, as adherence to program recommendations increased, participants lost an additional 1.8 kg by 6-months ($\gamma_{wtch,adh}$ = -1.787;

SE = 0.203; $p < 0.001$), controlling for covariates. Fig 2 displays the same path diagram with coefficients and factor loadings superimposed over directional arrows to aid interpretation.

Figure 2. Path diagrams with coefficients

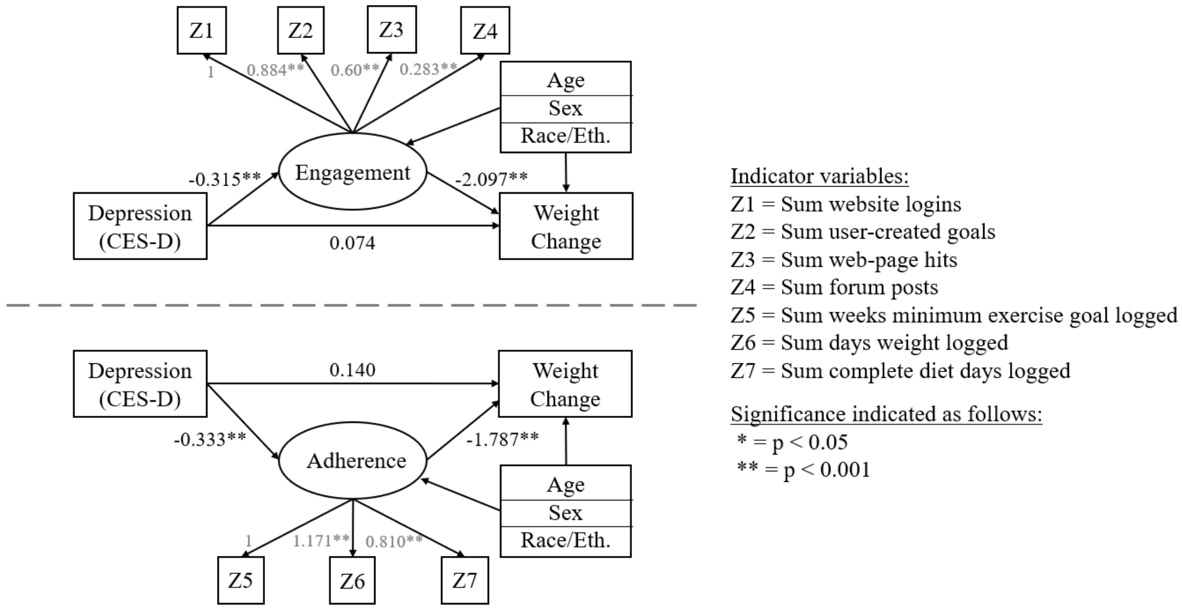

These results showed that the effects of baseline depression symptoms on weight change were completely mediated through the constructs of engagement and adherence in each model. The indirect effect of CES-D scores on 6-month weight change through engagement was 0.660 (SE = 0.170; $p < 0.001$). Likewise, the indirect effect of CES-D scores on 6-month weight change through adherence was 0.594 (SE = 0.148; $p < 0.001$). Rephrased in practical terms, participants with CES-D scores one standard deviation above the sample mean could expect to lose 0.59% less weight at 6 months than those with average CES-D scores when modeled through engagement, or 0.54% less weight when modeled through adherence, which are both significant (p’s < 0.01). These positive indirect effects on weight change would indicate attenuated weight loss, or possible weight gain, over 6 months by affected participants. Results did not meaningfully differ when using 12-month weight change values as the outcome.

Lastly, to describe the demographic control covariates in these twin pooled models: Age was the only consistently significant covariate across both engagement and adherence models, showing positive effects on engagement ($\gamma_{eng,age}$ = 0.037; SE = 0.008) and adherence ($\gamma_{adh,age}$ = 0.028; SE = 0.008), and negative effects on 6-month weight change in the engagement ($\gamma_{wtch,age}$ = -0.057; SE = 0.025) and adherence ($\gamma_{adh,age}$ = -0.083; SE = 0.026) models (all p's ≤ 0.001). Participant sex was significantly associated with 6-month weight loss in the engagement model ($\gamma_{wtch,sex}$ = -1.667; SE = 0.588; p = 0.005) and barely reached significance in the adherence model ($\gamma_{wtch,sex}$ = -1.190; SE = 0.604; p = 0.049), such that males tended to lose more weight than females in this study sample. Additionally, race/ethnicity was associated with reduced adherence only in that model ($\gamma_{adh,race}$ = -0.510; SE = 0.224; p = 0.023). These results were largely replicated when using percent weight change as the outcome; however, sex was no longer a significant predictor of weight change. This suggests that while males may have lost more absolute weight in this study than females, the relative reductions in percent weight did not statistically differ.

Discussion

The primary aim of this secondary analysis was to empirically test whether baseline depression symptoms exert undesirable influences on program engagement, adherence, and distal behavioral outcomes within a digital weight loss intervention. Results from this study fully support the guiding hypotheses and provide some quantitative estimates of the associations between depression symptoms and conduct within a digital weight management intervention.

This analysis shows that participants with mild to moderate depression symptoms (i.e., reporting CES-D scores < 16) can struggle to engage with website-based interventions and/or to

adhere to program recommendations, which can in turn have meaningful impacts on their behavior change outcomes. It is of growing importance for researchers to consider how depression may influence participant involvement in DBCIs, as population depression levels are climbing to unknown degrees since the COVID-19 pandemic, with some estimates documenting a >300% increase in adults reporting depression and anxiety symptoms between 2019-2021.(60, 61) National statistics tend to focus only on clinical, severe, or major depressive episodes, which likely underestimates the prevalence of people living with mild to moderate depression in the US.(62, 63)

This issue should be of particular interest for future digital weight management interventions, as it is known that depression symptoms and weight status are highly correlated, and that during the COVID-19 pandemic, those living with depression and anxiety experienced significantly greater weight gains than those without.(64-66) If these effects of depression symptoms remain unaddressed, future interventions may see reduced engagement, adherence, and reduced weight change. It would instead be prudent for future DBCIs to consider additional tailoring on depression symptoms and related factors measurable at baseline in tandem with sociodemographic and behavioral tailoring. Some possibilities might include tailoring the types of feedback messages sent by an intervention to avoid triggering negative attributions to failing goals,(67, 68) including lesson and review content tailored to depression and motivation at baseline to attempt to inoculate potential negative outcomes, relaxing goal standards in the early stages of an intervention to help participants to build initial self-efficacy, or possibly building in a 'time-out' feature if participants would want to take a short break from a program without worrying about failing goals in the interim. In a meta-synthesis of user experiences with DBCI's for depression and anxiety, Knowles *et al*. synthesized themes that computerized tailoring should

be sensitive to 'Who I am' for personalized relevance, as well as to 'How I feel' to be appropriate for those experiencing low moods and low motivation typical of depression.(69)

This analysis has several limitations. First, due to the nature of data available, there were limited system usage indicators to measure program adherence and engagement. While the original intent of this analysis was to model both engagement and adherence simultaneously, the two LVs were too highly correlated to model meaningful results, despite efforts to distinguish the page-hits indicator from all self-monitoring activities. This is likely because all behavioral self-monitoring in LNPA required manual logging using the IWL website. DBCIs using passive data collection methods such as wearable activity trackers to measure participant adherence to exercise goals may be more capable of comparing these LVs within the same model. Second, the LNPA study functionality which allowed participants to change dietary monitoring strategies and physical activity goal intensity *ad libitum* during the intervention changed the standards for how data were recorded (e.g., one dietary format had users manually log all calories they consumed, while another was assigned to a 'meal plan' format that automatically recorded preset menu items with calories and requested users note any deviations from that plan) and created challenges for consistently operationalizing longitudinal indicators of program adherence. This resulted in dietary and physical activity adherence indicators varying over time for a large number of participants and necessitated for all indicators to be brought into the same coarse scale for this analysis. This also may have contributed to the adherence LV contributing less unique predictive capability than anticipated, as participants likely to be recorded as having higher adherence would necessarily need to log into the IWL website more often. Future examinations these relationships using longitudinal methods and measures will be valuable to understand if and how these relationships may vary over time. Third, the LNPA study sample lacked racial and

gender diversity with a majority proportion of white female participants, which is unfortunately not uncommon in DBCI research.(46, 47)

Strengths of this analysis include the high-fidelity, automated program delivery across all users within a real-world setting and extensive collection of objective participant usage metrics. Additionally, the parent study included a majority of participants who had not achieved a Bachelor's degree or equivalent, approximately 25% male participants, included many older adult participants with a wide range of BMIs, and also included many adults living with comorbidities such as diabetes all recruited within primary care settings.

Conclusion

This analysis empirically showed that depression symptoms can negatively influence distal weight loss within a website-based digital weight management intervention, and that this effect is fully mediated through engagement and adherence. This finding may point to an important factor contributing to observed declines in participant engagement over time in DBCIs. The field of digital behavior change interventions would greatly benefit from future research examining if tailoring digital interventions on baseline depression levels can help ameliorate these negative relationships, and whether other psychological factors may be influencing participant engagement, adherence, and overall success in these interventions.

Acknowledgements

The authors would like to thank the faculty and staff of the UNC Weight Research Program.

References

1. Rivera J, McPherson A, Hamilton J, Birken C, Coons M, Iyer S, et al. Mobile apps for weight management: a scoping review. JMIR mHealth and uHealth. 2016;4(3):e87.
2. Mangieri CW, Johnson RJ, Sweeney LB, Choi YU, Wood JC. Mobile health applications enhance weight loss efficacy following bariatric surgery. Obesity Research & Clinical Practice. 2019;13(2):176-9.
3. Shaw RJ, Bosworth HB, Silva SS, Lipkus IM, Davis LL, Sha RS, Johnson CM. Mobile Health Messages Help Sustain Recent Weight Loss. The American Journal of Medicine. 2013;126(11):1002-9.
4. Flores Mateo G, Granado-Font E, Ferré-Grau C, Montaña-Carreras X. Mobile Phone Apps to Promote Weight Loss and Increase Physical Activity: A Systematic Review and Meta-Analysis. J Med Internet Res. 2015;17(11):e253.
5. Vandelanotte C, Müller AM, Short CE, Hingle M, Nathan N, Williams SL, et al. Past, Present, and Future of eHealth and mHealth Research to Improve Physical Activity and Dietary Behaviors. Journal of Nutrition Education and Behavior. 2016;48(3):219-28.e1.
6. Statistics NCfH. Obesity and Overweight https://www.cdc.gov/nchs/fastats/obesity-overweight.htm: Centers for Disease Control and Prevention; 2021 [Percent of adults aged 20 and over with obesity: 42.5% (2017-8)

Percent of adults aged 20 and over with overweight, including obesity: 73.6% (-8)

Source: Prevalence of Overweight, Obesity, and Severe Obesity Among Adults Aged 20 and Over: United States, 1960-2 Through 2017-8].
7. Fryar CD, Carroll MD, Ogden CL. Prevalence of overweight, obesity, and severe obesity among children and adolescents aged 2–19 years: United States, 1963–1965 through 2015–2016. 2018.
8. Perski O, Blandford A, West R, Michie S. Conceptualising engagement with digital behaviour change interventions: a systematic review using principles from critical interpretive synthesis. Transl Behav Med. 2017;7(2):254-67.
9. Short CE, DeSmet A, Woods C, Williams SL, Maher C, Middelweerd A, et al. Measuring Engagement in eHealth and mHealth Behavior Change Interventions: Viewpoint of Methodologies. J Med Internet Res. 2018;20(11):e292.
10. Pham Q, Graham G, Carrion C, Morita PP, Seto E, Stinson JN, Cafazzo JA. A Library of Analytic Indicators to Evaluate Effective Engagement with Consumer mHealth Apps for Chronic Conditions: Scoping Review. JMIR Mhealth Uhealth. 2019;7(1):e11941.
11. Christensen H, Mackinnon A. The Law of Attrition Revisited. J Med Internet Res. 2006;8(3):e20.
12. Eysenbach G. The Law of Attrition. J Med Internet Res. 2005;7(1):e11.
13. Yeager CM, Benight CC. If we build it, will they come? Issues of engagement with digital health interventions for trauma recovery. Mhealth. 2018;4:37-.
14. Lie SS, Karlsen B, Oord ER, Graue M, Oftedal B. Dropout From an eHealth Intervention for Adults With Type 2 Diabetes: A Qualitative Study. J Med Internet Res. 2017;19(5):e187.
15. Price M, Gros DF, McCauley JL, Gros KS, Ruggiero KJ. Nonuse and dropout attrition for a web-based mental health intervention delivered in a post-disaster context. Psychiatry. 2012;75(3):267-84.
16. Sabate E. World Health Organization. Adherence to Long-term Therapies: Evidence for Action. http://www.who.int/chp/knowledge/publications/adherence_full_report.pdf: World Health Organization; 2003.
17. Sieverink F, Kelders SM, van Gemert-Pijnen JEWC. Clarifying the Concept of Adherence to eHealth Technology: Systematic Review on When Usage Becomes Adherence. J Med Internet Res. 2017;19(12):e402.

18. Christensen H, Griffiths KM, Farrer L. Adherence in internet interventions for anxiety and depression: systematic review. Journal of medical Internet research. 2009;11(2):e13.
19. Donkin L, Christensen H, Naismith SL, Neal B, Hickie IB, Glozier N. A Systematic Review of the Impact of Adherence on the Effectiveness of e-Therapies. J Med Internet Res. 2011;13(3):e52.
20. Jakob R, Harperink S, Rudolf AM, Fleisch E, Haug S, Mair JL, et al. Factors Influencing Adherence to mHealth Apps for Prevention or Management of Noncommunicable Diseases: Systematic Review. J Med Internet Res. 2022;24(5):e35371.
21. Sieverink F, Kelders SM, van Gemert-Pijnen JE. Clarifying the Concept of Adherence to eHealth Technology: Systematic Review on When Usage Becomes Adherence. J Med Internet Res. 2017;19(12):e402.
22. Oppezzo MA, Stanton MV, Garcia A, Rigdon J, Berman JR, Gardner CD. To Text or Not to Text: Electronic Message Intervention to Improve Treatment Adherence Versus Matched Historical Controls. JMIR Mhealth Uhealth. 2019;7(4):e11720.
23. Thomas JG, Bond DS. Behavioral response to a just-in-time adaptive intervention (JITAI) to reduce sedentary behavior in obese adults: Implications for JITAI optimization. Health Psychology. 2015;34(Suppl):1261-7.
24. Turner-McGrievy GM, Dunn CG, Wilcox S, Boutté AK, Hutto B, Hoover A, Muth E. Defining Adherence to Mobile Dietary Self-Monitoring and Assessing Tracking Over Time: Tracking at Least Two Eating Occasions per Day Is Best Marker of Adherence within Two Different Mobile Health Randomized Weight Loss Interventions. J Acad Nutr Diet. 2019;119(9):1516-24.
25. Wang J, Sereika SM, Chasens ER, Ewing LJ, Matthews JT, Burke LE. Effect of adherence to self-monitoring of diet and physical activity on weight loss in a technology-supported behavioral intervention. Patient Prefer Adherence. 2012;6:221-6.
26. Wing RR, Phelan S, Tate D. The role of adherence in mediating the relationship between depression and health outcomes. Journal of psychosomatic research. 2002;53(4):877-81.
27. Pourzanjani A, Quisel T, Foschini L. Adherent Use of Digital Health Trackers Is Associated with Weight Loss. PLoS One. 2016;11(4):e0152504-e.
28. Butryn ML, Godfrey KM, Martinelli MK, Roberts SR, Forman EM, Zhang F. Digital self-monitoring: Does adherence or association with outcomes differ by self-monitoring target? Obesity Science & Practice. 2020;6(2):126-33.
29. Noh J-W, Kwon YD, Park J, Kim J. Body mass index and depressive symptoms in middle aged and older adults. BMC public health. 2015;15(1):1-7.
30. Istvan J, Zavela K, Weidner G. Body weight and psychological distress in NHANES I. International Journal of Obesity and Related Metabolic Disorders: Journal of the International Association for the Study of Obesity. 1992;16(12):999-1003.
31. Breymeyer KL, Lampe JW, McGregor BA, Neuhouser ML. Subjective mood and energy levels of healthy weight and overweight/obese healthy adults on high-and low-glycemic load experimental diets. Appetite. 2016;107:253-9.
32. Kerr J, Patrick K, Norman G, Stein MB, Calfas K, Zabinski M, Robinson A. Randomized control trial of a behavioral intervention for overweight women: impact on depressive symptoms. Depression and anxiety. 2008;25(7):555-8.
33. Singh G, Jackson C, Dobson A, Mishra G. Bidirectional association between weight change and depression in mid-aged women: a population-based longitudinal study. International Journal of Obesity. 2014;38(4):591-6.
34. Bossen D, Buskermolen M, Veenhof C, de Bakker D, Dekker J. Adherence to a web-based physical activity intervention for patients with knee and/or hip osteoarthritis: a mixed method study. Journal of medical Internet research. 2013;15(10):e223.

35. Postel MG, de Haan HA, Ter Huurne ED, van der Palen J, Becker ES, de Jong CA. Attrition in web-based treatment for problem drinkers. Journal of medical Internet research. 2011;13(4):e117.
36. Al-Asadi AM, Klein B, Meyer D. Pretreatment attrition and formal withdrawal during treatment and their predictors: an exploratory study of the anxiety online data. Journal of medical Internet research. 2014;16(6):e152.
37. Neve MJ, Collins CE, Morgan PJ. Dropout, nonusage attrition, and pretreatment predictors of nonusage attrition in a commercial Web-based weight loss program. Journal of Medical Internet Research. 2010;12(4):e69.
38. Nicholas J, Proudfoot J, Parker G, Gillis I, Burckhardt R, Manicavasagar V, Smith M. The ins and outs of an online bipolar education program: a study of program attrition. Journal of medical Internet research. 2010;12(5):e57.
39. Richardson A, Graham AL, Cobb N, Xiao H, Mushro A, Abrams D, Vallone D. Engagement promotes abstinence in a web-based cessation intervention: cohort study. Journal of medical Internet research. 2013;15(1):e14.
40. Habibović M, Cuijpers P, Alings M, van der Voort P, Theuns D, Bouwels L, et al. Attrition and Adherence in a Web-Based Distress Management Program for Implantable Cardioverter Defibrillator Patients (WEBCARE): Randomized Controlled Trial. J Med Internet Res. 2014;16(2):e52.
41. Hebert EA, Vincent N, Lewycky S, Walsh K. Attrition and adherence in the online treatment of chronic insomnia. Behavioral sleep medicine. 2010;8(3):141-50.
42. Haukkala A, Uutela A, Vartiainen E, Mcalister A, Knekt P. Depression and smoking cessation: the role of motivation and self-efficacy. Addictive behaviors. 2000;25(2):311-6.
43. Linde JA, Jeffery RW, Levy R, Sherwood NE, Utter J, Pronk N, Boyle R. Binge eating disorder, weight control self-efficacy, and depression in overweight men and women. International Journal of obesity. 2004;28(3):418-25.
44. Tate DF, Kraschnewski JL, Martinez C, Diamond M, Veldheer S, Hwang KO, et al. A cluster-randomized controlled trial of automated internet weight-loss programs in primary care: Role of automated provider feedback. Obesity. 2022;30(12):2363-75.
45. Roberts AL, Fisher A, Smith L, Heinrich M, Potts HW. Digital health behaviour change interventions targeting physical activity and diet in cancer survivors: a systematic review and meta-analysis. Journal of Cancer Survivorship. 2017;11(6):704-19.
46. Hutchesson MJ, Rollo ME, Krukowski R, Ells L, Harvey J, Morgan PJ, et al. eHealth interventions for the prevention and treatment of overweight and obesity in adults: a systematic review with meta-analysis. Obesity reviews. 2015;16(5):376-92.
47. Sherrington A, Newham J, Bell R, Adamson A, McColl E, Araujo-Soares V. Systematic review and meta-analysis of internet-delivered interventions providing personalized feedback for weight loss in overweight and obese adults. obesity reviews. 2016;17(6):541-51.
48. Herrero J, Meneses J. Short Web-based versions of the perceived stress (PSS) and Center for Epidemiological Studies-Depression (CESD) Scales: A comparison to pencil and paper responses among Internet users. Computers in Human Behavior. 2006;22(5):830-46.
49. Hurley AR. Assessing Depression and Attributional Styles as Determinants of Engagement in Digital Behavior Change Interventions: The University of North Carolina at Chapel Hill; 2023.
50. Nezami BT, Hurley L, Power J, Valle CG, Tate DF. A pilot randomized trial of simplified versus standard calorie dietary self-monitoring in a mobile weight loss intervention. Obesity. 2022;30(3):628-38.
51. Von Ruesten A, Illner A, Buijsse B, Heidemann C, Boeing H. Adherence to recommendations of the German food pyramid and risk of chronic diseases: results from the EPIC-Potsdam study. European journal of clinical nutrition. 2010;64(11):1251-9.
52. Bollen K. Chapter 4: Simultaneous Equation Models. In: NA, editor. Foundations of Structural Equation Models. 1. NA: NA; 2020 (in press). p. NA.

53. Bollen KA. Structural equations with latent variables: John Wiley & Sons; 1989.
54. Bollen KA, Pearl J. Eight myths about causality and structural equation models. Handbook of causal analysis for social research: Springer; 2013. p. 301-28.
55. Rosseel Y. lavaan: An R Package for Structural Equation Modeling. 2012. 2012;48(2):36.
56. Rosseel TDJaSPaAMSaY. semTools: Useful tools for structural equation modeling. R package version 0.5-5 https://CRAN.R-project.org/package=semTools2021 [cited 2022.
57. Rubin DB. Multiple Imputation for Nonresponse in Surveys. New York: John Wiley & Sons, Inc.; 1987.
58. van Buuren S, Groothuis-Oudshoorn K. mice: Multivariate Imputation by Chained Equations in R. Journal of Statistical Software. 2011;45(3):1 - 67.
59. Rosseel Y. lavaan: An R Package for Structural Equation Modeling. Journal of Statistical Software. 2012;48(2):1 - 36.
60. Pfefferbaum B, North CS. Mental health and the Covid-19 pandemic. New England Journal of Medicine. 2020;383(6):510-2.
61. DeAngelis T. Depression and anxiety escalate during COVID. Monitor on Psychology: American Psychology Association; 2021 [Available from: https://www.apa.org/monitor/2021/11/numbers-depression-anxiety.
62. CDC. Prevalence of Depression Among Adults Aged 20 and Over: United States, 2013-2016 https://www.cdc.gov/nchs/products/databriefs/db303.htm: Centers for Disease Control and Prevention; 2018 [
63. NIMH. Major Depression https://www.nimh.nih.gov/health/statistics/major-depression: National Institutes of Mental Health; 2019 [updated February 2019.
64. Luppino FS, de Wit LM, Bouvy PF, Stijnen T, Cuijpers P, Penninx BW, Zitman FG. Overweight, obesity, and depression: a systematic review and meta-analysis of longitudinal studies. Archives of general psychiatry. 2010;67(3):220-9.
65. Khubchandani J, Price JH, Sharma S, Wiblishauser MJ, Webb FJ. COVID-19 pandemic and weight gain in American adults: A nationwide population-based study. Diabetes Metab Syndr. 2022;16(1):102392.
66. Bhutani S, vanDellen MR, Cooper JA. Longitudinal Weight Gain and Related Risk Behaviors during the COVID-19 Pandemic in Adults in the US. Nutrients. 2021;13(2):671.
67. Tolli AP, Schmidt AM. The role of feedback, causal attributions, and self-efficacy in goal revision. Journal of Applied Psychology. 2008;93(3):692.
68. Bandura A, Locke EA. Negative self-efficacy and goal effects revisited. Journal of applied psychology. 2003;88(1):87.
69. Knowles SE, Toms G, Sanders C, Bee P, Lovell K, Rennick-Egglestone S, et al. Qualitative meta-synthesis of user experience of computerised therapy for depression and anxiety. PLoS One. 2014;9(1):e84323.

Supporting information

S1 Fig. Structural equation model path diagrams

S2 Fig. Path diagrams with coefficients

Fig 1

Click here to access/download;Figure;Fig 1.tif

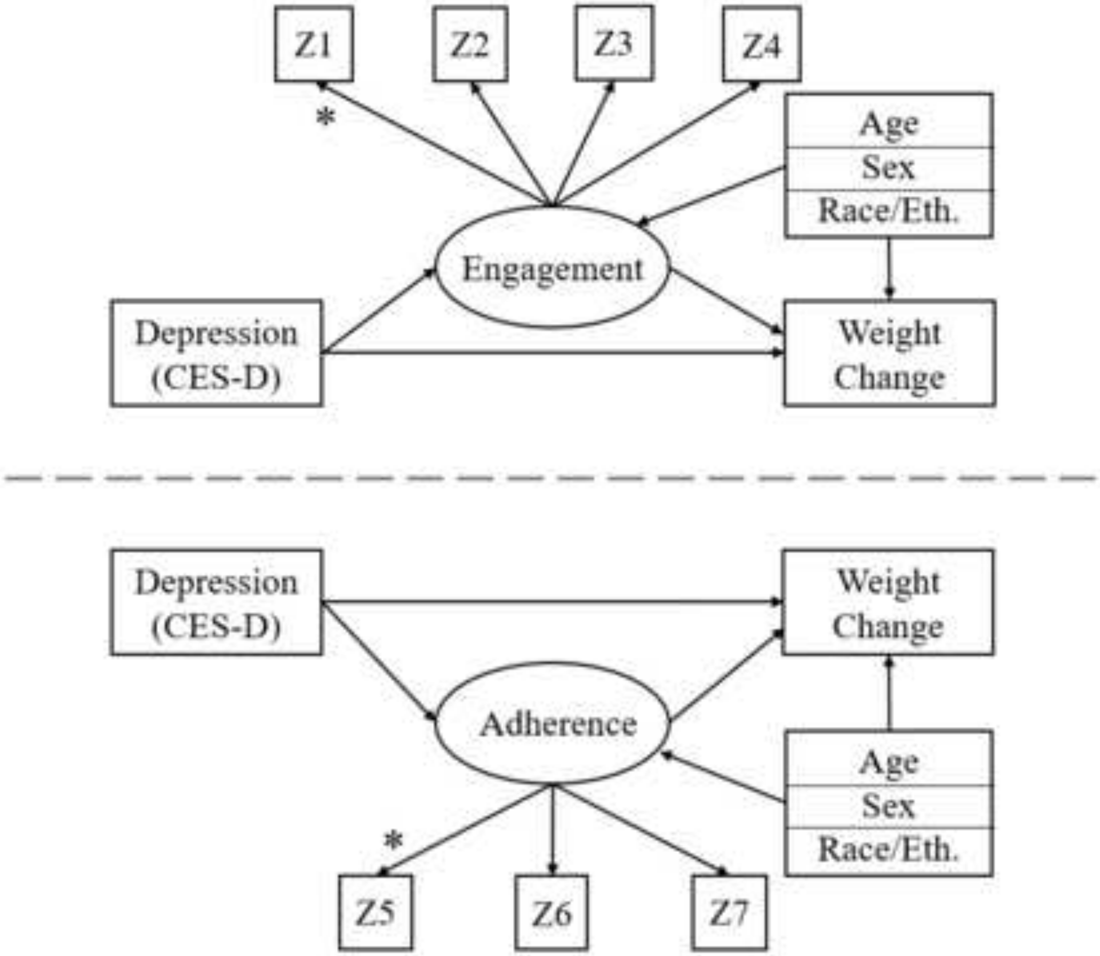

Indicator variables:
Z1 = Sum website logins
Z2 = Sum user-created goals
Z3 = Sum web-page hits
Z4 = Sum forum posts
Z5 = Sum weeks minimum exercise goal logged
Z6 = Sum days weight logged
Z7 = Sum complete diet days logged

* = loading variable

Fig 2

Click here to access/download;Figure;Fig 2.tif

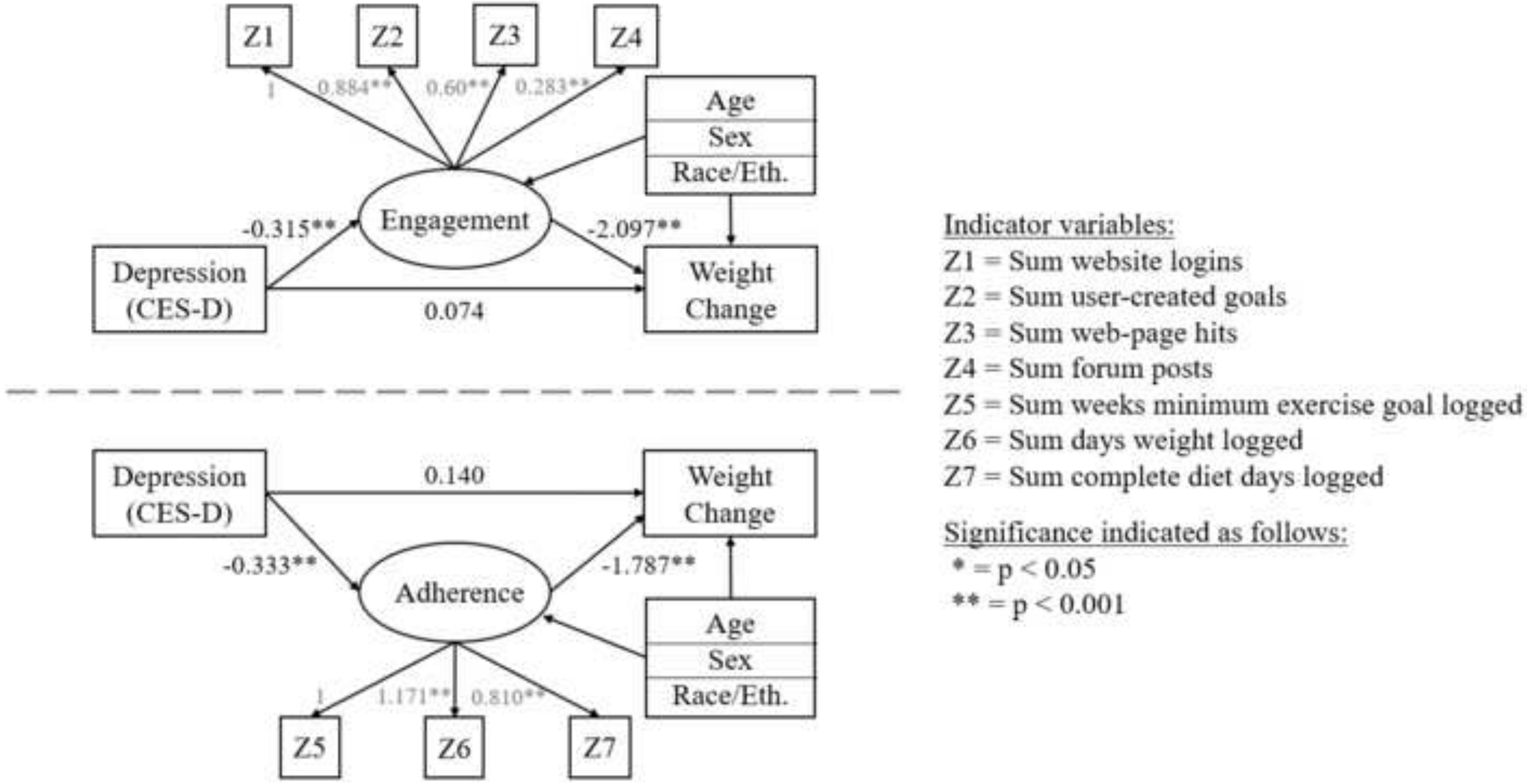

Supplement: Attachment — Submitted filename: Reviewer feedback_9-9-2024.pdf [file pdig.0000766.s002.pdf]
