# Supplementary material for: Measuring the influence of depressive symptoms on engagement, adherence, and weight loss in an eHealth intervention
Source: PLOS Digit Health. 2025 Mar 25;4(3):e0000766. doi: 10.1371/journal.pdig.0000766 (PMC11936179; doi:10.1371/journal.pdig.0000766)
Supplement: Attachment — Submitted filename: Response to Reviewers.docx [file pdig.0000766.s003.docx]

Hello all, I organized responses to each item sent from the Editor and Reviewers written in this blue Times New Roman font. Unfortunately, the attached PDF with reviewer comments did not specify the source of each highlighted comment, so I have copy/pasted them with responses in the order received at the bottom of this document. Thank you all for taking the time to review this manuscript.

Editor Comments:

1. Your current Financial Disclosure states, “T32 Cancer Health Disparities Training Grant from the National Cancer Institute of the National Institutes of Health”. However, your funding information on the submission form indicates that you received funding from “Center for Biomedical Informatics and Information Technology, National Cancer Institute” and Grant Recipient "Dr. Lex Hurley". Please indicate by return email the full and correct funding information for your study and confirm the order in which funding contributions should appear. Please be sure to indicate whether the funders played any role in the study design, data collection and analysis, decision to publish, or preparation of the manuscript.

Thank you for this comment. I have updated our Financial Disclosure statement based on these recommendations and included it in the cover letter and online form.

2. In the online submission form, you indicated that "The authors do not have permission to share raw study data due to requirements to protect the privacy of participants, in accordance with their informed consent. However, de-identified data used in this specific analysis and/or output files related to this analysis will be made available upon request to the corresponding author."[…]

My apologies, I was unaware that this was unsatisfactory for the journal. I have uploaded the de-identified dataset to a secure repository affiliated with our university and provided the information and link in the cover letter and online form.

3. We notice that your figures are included in the manuscript file. Please remove them as there is already Figures uploaded in the file inventory. Also remove the citation "Supporting information S1 Fig. Structural equation model path diagrams S2 Fig. Path diagrams with coefficients" listed in the manuscript after the references list.

Completed.

_____________________________________________________________________________________


[Note: HTML markup is below. Please do not edit.]

Reviewers' comments:

Reviewer's Responses to Questions

<font color="black"><b>Comments to the Author</b><br><br>
1. Does this manuscript meet PLOS Digital Health’s <a href="<https://journals.plos.org/digitalhealth/s/journal-information#loc-criteria-for-publication>" target="_blank">publication criteria</a>? Is the manuscript technically sound, and do the data support the conclusions? The manuscript must describe methodologically and ethically rigorous research with conclusions that are appropriately drawn based on the data presented.</font>


Reviewer #1: Partly

Reviewer #2: Yes

Reviewer #3: No

--------------------

<font color="black">2. Has the statistical analysis been performed appropriately and rigorously?</font>

Reviewer #1: N/A

Reviewer #2: Yes

Reviewer #3: I don't know

--------------------

<font color = "black">3. Have the authors made all data underlying the findings in their manuscript fully available (please refer to the Data Availability Statement at the start of the manuscript PDF file)?<br><br> The <a href="<https://journals.plos.org/digitalhealth/s/data-availability>" target="_blank">PLOS Data policy</a> requires authors to make all data underlying the findings described in their manuscript fully available without restriction, with rare exception. The data should be provided as part of the manuscript or its supporting information, or deposited to a public repository. For example, in addition to summary statistics, the data points behind means, medians and variance measures should be available. If there are restrictions on publicly sharing data—e.g. participant privacy or use of data from a third party—those must be specified.</font>

Reviewer #1: No

Reviewer #2: No

Reviewer #3: Yes

--------------------

<font color = "black">4. Is the manuscript presented in an intelligible fashion and written in standard English?<br/><br/>PLOS Digital Health does not copyedit accepted manuscripts, so the language in submitted articles must be clear, correct, and unambiguous. Any typographical or grammatical errors should be corrected at revision, so please note any specific errors here.</font>

Reviewer #1: No

Reviewer #2: Yes

Reviewer #3: No

--------------------

<font color = "black">5. Review Comments to the Author<br/><br/>Please use the space provided to explain your answers to the questions above. You may also include additional comments for the author, including concerns about dual publication, research ethics, or publication ethics. (Please upload your review as an attachment if it exceeds 20,000 characters)</font>

Reviewer #1: Dear Authors,
Your research has not found any new cofounder of depression. Weight gain due to depression and vice versa are the known facts now.
Therefore, I am rejecting it for publication.
Thank you.

In the introduction, I cite several studies showing that depression, weight status, and weight gain are well-known to be associated with one another, as this is indeed known. However, what *is* novel about this study is that it examines the ways that depression can impact participant engagement and adherence in an eHealth digital behavior change intervention (DBCI) which is shown to overall reduce the effectiveness of this intervention to promote weight management. As DBCI methodology is distinct from traditional in-person interventions, this information is valuable. Moreover, many studies examining engagement and adherence use single indicators for these constructs via regression, which assumes that one measure of these constructs is as valid as any other measure. This study also addresses this limitation by simultaneously measuring multiple indicators of each construct to provide more secured estimates. Based on editor and reviewer feedback, I have expanded on previous research via depression and traditional in-person weight management studies to better differentiate this analysis from previous work.

Reviewer #2: Review of Manuscript: "Measuring the influence of depressive symptoms on engagement, adherence, and weight loss in a 6-month eHealth intervention"

This manuscript addresses a pertinent issue in the field of digital health, specifically the influence of depressive symptoms on engagement, adherence, and subsequent weight loss in an eHealth intervention. Given the increasing prevalence of digital behavior change interventions for weight management, understanding the psychological factors that impact their effectiveness is both timely and relevant. The use of structural equation modeling (SEM) to explore the relationships between depression, engagement, adherence, and weight outcomes is a strength of the study, as it allows for a nuanced understanding of these complex interactions. However, there are areas where clarity, methodological detail, and interpretation of results could be improved to enhance the manuscript's overall contribution to the field.

Abstract:
The abstract does address the core components (background, methods, results, discussion), but the methods section could use more detail to fully explain the research approach. The results section lacks quantitative results (e.g., statistical significance values), which are often expected in scientific abstracts. For instance, line 36, the phrase “positive indirect association” might confuse some readers without further clarification that it leads to less weight loss or potential weight gain. The discussion provides a broad statement but lacks actionable insights or suggestions, which could strengthen the conclusion.

Thank you for these notes. I have attempted to bolster the methods and discussion sections of the abstract, and provided p-values for each of the results interpretation statements. Please let me know if you would prefer the coefficient values in addition to the p-values to provide some additional indicators of associations (though they may lack interpretability).

Introduction:
The introduction provides a solid background, defining key concepts and linking them to previous research. However, the review of literature might benefit from being a bit more focused, as the numerous references to different studies could overwhelm readers without clear synthesis. The hypotheses are clearly stated at the end of the introduction, outlining the goals of the analysis. This sets the stage for the research question and the methodology used to address it.
Suggestions: The 'Law of Attrition' is referenced as an important concept in DBCI research, but a brief explanation of why this law is relevant to the current study (beyond novelty) might strengthen the argument.
The discussion around the possible role of depression as a contributor to disengagement/dropout could be expanded to explain why this is the case (e.g., motivation, fatigue, psychological barriers).
Some of the references to past studies could be more tightly integrated into the central narrative to reduce the feeling of listing various sources. It would be beneficial to show how they collectively lead to the formulation of the study's hypotheses.

Thank you for this feedback. I have expanded on the Law of Attrition section of the Introduction to provide some greater detail on the processes that Eysenbach proposed, including replacement and disenchantment discontinuance. To address other reviewers’ feedback, I have also rewritten a portion of the introduction to provide more detail of what is known regarding depression and weight management in traditional interventions, then in bring the focus back to DBCIs by looping this relevance back to the disenchantment discontinuance from the Law of Attrition. I hope these edits have sufficiently strengthened the study’s Introduction.

Methods:
The Methods section provides a thorough and structured description of the study's design, dataset, measures, and analytical approach, making it very clear for replication or further understanding of the research process. Overall, the methods are strong, comprehensive, and appropriate for the study objectives.

Thank you for this feedback.

Results:
The section begins by clearly explaining the fit of the simultaneous equation models (SEM) using various fit indices (RMSEA, CFI, TLI, SRMR), which is a good approach. The use of multiple indices provides a comprehensive picture of model performance. It could be improved by briefly explaining why chi-square (χ²) was significant despite the other fit indices indicating good fit. A more nuanced discussion of the limitations of χ² with small sample sizes could enhance the reader’s understanding.
The statistical results are well-presented with relevant coefficients, standard errors (SE), and p-values, providing transparency. The text describes the absence of direct effects and highlights the mediation role of engagement and adherence in the relationship between depression symptoms and weight change, which is critical. Consider adding a more intuitive explanation of the indirect effects. For instance, explaining how engagement and adherence mediate weight change could be framed more clearly to emphasize the importance of these constructs.
The key results are summarized efficiently in Table 3, and the significant effects of age, sex, and race/ethnicity on engagement, adherence, and weight change are appropriately addressed. The section could benefit from clearer transitions between the main results (e.g., engagement/adherence) and the secondary findings (e.g., demographic effects). This would help maintain focus on the primary hypotheses and findings while still reporting on important covariates.
The indirect effects of CES-D scores on weight change are explained with practical examples, such as a standard deviation increase in CES-D scores leading to specific percentages of reduced weight loss. These practical interpretations are excellent, but the narrative could expand on why these indirect effects are meaningful in a real-world context. For example, explaining how even a modest decrease in weight loss due to lower engagement/adherence could have clinical implications might strengthen the impact of the findings.

Thank you for these notes. I have revised the sentence related to significant Chi-square values to more directly explain why these values were significant, rather than alluding to the mechanisms. I have also revised the practical explanations of the indirect effects for engagement and adherence, with separate sentences for each interpretation. Regarding the final comment here about expanding on these indirect effects in a real-world context, I have revised part of the discussion section to try and provide some more clinically-relevant interpretations of these results, which I hope are satisfactory to address this comment.

Discussion:
The discussion highlights key strengths, including the high-fidelity delivery and the objective collection of participant usage metrics, which are important aspects of the study. The strengths could be expanded by emphasizing the novelty or significance of the findings in digital health research. For instance, discussing how these insights add to the understanding of the impact of depression on digital health interventions would underscore the contribution of the study.

Thank you for these last comments. I have worked to expand on some additional elements of the strengths, as indicated, and emphasized the novel contribution of this study to the digital behavior change intervention literature within the conclusion. Your feedback has been exceptionally helpful to strengthen this manuscript and prepare it for publication.

Reviewer #3: I think your research questions are very important hence, I didn't reject your draft although I have serious concerns about the fundamentals of methodology, introduction, and language of draft overall (e.g., full of jargon) that requires further work on clarification and correction. I would prefer to give you a second chance in writing the draft given that you were honest in sharing your limitations and have important research questions maybe you can do modifications or rectify the problems or add more information and resubmit the draft. If you have omitted information that may have led to us not understanding something in your study, this is your chance to clarify and add that to improve your draft. I highly recommend that you consult with the clinical psychologist in your team about the psychological constructs concern for example, she could have helpful insights. Please find my specific comments on the attached pdf file for your review. Hope they are helpful.

Thank you for taking the time to review our manuscript. To address each of your comments in order, I should begin by noting that the Methods section is intentionally comprehensive in this study to provide clear descriptions for readers to facilitate replication of study analyses, if desired. My coauthors and I have reviewed these sections in light of comments from the editor and reviewers and do not believe there is excessive usage of unnecessary terms or jargon. I am unclear as to what you are inferring via the “omitted information that may have led us to not understanding something in your study” passage in this comment, as I have taken express interest in clearly operationalizing and defining all aspects and limitations related to the data and conclusions of this study. Based on editor and reviewer comments, we have expanded on some sections related to previous research via traditional in-person weight management interventions and depressive symptoms to provide some additional context for readers. Your referred psychological constructs concern is addressed in the pasted comments from the PDF section below.

--------------------

<font color = "black">6. PLOS authors have the option to publish the peer review history of their article (<a href="<https://journals.plos.org/digitalhealth/s/editorial-and-peer-review-process#loc-peer-review-history>" target="_blank">what does this mean?</a>). If published, this will include your full peer review and any attached files.<br><br>

<b>Do you want your identity to be public for this peer review?</b> If you choose “no”, your identity will remain anonymous but your review may still be made public.<br><br>

For information about this choice, including consent withdrawal, please see our <a href="<https://www.plos.org/privacy-policy>" target="_blank">Privacy Policy</a>.</font>


Reviewer #1: Yes: Subhagata Chattopadhyay

Reviewer #2: No

Reviewer #3: No

--------------------
_________________________________________________________________________________

**Comments pasted from the attached manuscript PDF:**

Originality - there is need for this research, but there has been research on depression and obesity treatment or weight management programs at least in traditional in person that should have been included in forming the hypotheses and in guiding the conclusions. In my opinion the introduction section and the conclusion section are neglecting that

High importance and broad interest to community of researchers, engineers and clinicians working in the field of digital health

High methodological rigor and ethical standards

Substantial evidence for its conclusions

Clearly outlined utility and accessibility for the broader community

Follow appropriate standards and practice of open science

Thank you for these notes. I have revised and expanded on previous research in the introduction to better describe the state of the state of the research regarding depression and adherence in traditional weight loss interventions. I have also expanded on the discussion section, returning additional acknowledgement to traditional in-person weight management intervention research.

-----

not sure about "anecdotally" here because previous research was done in this area at least in traditional weight management programs

Thank you for this comment. My phrasing in this sentence referred specifically to digital weight management interventions, which have very different needs and requirements than traditional in-person weight management interventions, and also have not yet had the influences of depression quantified as have done in this study. Based editor and reviewer comments, I have added an additional paragraph briefly expanding on what is known regarding depression and weight in traditional interventions before returning the focus to digital interventions.

-----

I think there is need to parse out the effect of each depressive symptom or do analysis controlling for some somatic symptoms versus non-somatic symptoms and screening participants for eating disorders because there will be differences in their effect on weight change and potential confounding factors for research

As the CES-D is a validated scale to measure depressive symptoms, breaking it apart in this manner would compromise its validity and reliability, and also necessitate an entirely different analysis than this current study. Additionally, all participants were screened for eating disorders prior to participation in the parent LNPA study, as described in the cited primary outcomes paper.

-----

[regarding the use of social forum posts as an indicator variable] this feature could be affected by social desirability factors or privacy desires aside from real enagagment in the program

This is plausible. However, sum social forum posts over 6 months is used as but one of four indicator variables informing the engagement latent variable (LV) for analysis. Additionally, the relatively small factor loading of 0.283 (p < 0.001) compared to the other indicator variables (each ≥0.6, p’s < 0.001) indicate that even if such confounding influences were affecting some participants in this program, it is exceedingly unlikely that they would exert any meaningful bias to alter the observed relationships or conclusions.

-----

I think explaining the functions of these tests and avoiding jargon here is essential for reviewers/readers who are not familiar with the jargon to understand the basis of statistical analysis and your choices for procedures

These are common goodness-of-fit tests for structural equation modeling, similar to F-tests, AIC, and BIC in typical regression. These are typically not individually explained to readers in each publication, as to do so would deviate from the primary focus of each study and increase the level of requisite jargon to explain them, which you noted in your overall comments. To address another comment, we have revised the section reporting the significant χ^2^ tests to better explain the likely reason for its significance. Beyond this, each reported result is interpreted as indicative of good or fair fit for readers, so they may have some confidence in the accuracy of the following model results.

-----

I think there is a wellknown sex difference in the prevlance of depression between males and females in ways that could affect this observation which should be noted in describing results.

While you are correct that there is a known difference in prevalence of depressive symptoms based on sex, describing how sex may predict depression symptoms in this sample is not of focal interest in this analysis. Rather, sex is used as a control covariate for engagement, adherence, and weight change to help ensure that the observed direct and indirect effects are attributable to changes in depressive symptoms and not demographics like sex. Therefore, the relationship you stated does not confound the present analyses.

-----

be careful with these statements as you didn't define nor differentiated what is race and what is ethnicity and that would bring into question possible other demographic factors that could inter-sect and interplay with them to cloud this and deem the statements misleading rather than informative. e.g., an educated high income hispanic or african american is different than a low income person of the same race or ehnicity. these kinds of discussions should be taken very seriousely and studied thoroughly

This comment’s basis in intersectionality theory is appreciated, and I agree such statements should be seriously considered before making any such types of explanatory or causal statements. However, the referred sentence is a simple interpretation of significant direct effects among control covariates within a larger model, and is largely used as an aside to facilitate reader interpretation of associations among this study sample (since similar interpretations were given for all other covariates in the SEM), rather than making claims of demographic drivers of success in these types of digital interventions.

-----

this statement needs better phrasing or linguistic clarification. It is not clear what you mean by "tailoring". I think you mean personalization or personal tailoring to patient's needs?. better to rewrite it more clearly.

“Tailoring” is a commonly applied term in digital behavior change intervention and communications research referring to the extent that a given user’s intervention messages and content are personalized based on measured characteristics about them. I have added the following excerpt to this sentence to aid in clarification:

“It would instead be prudent for future DBCIs to consider additional tailoring (i.e., personalizing intervention messaging and content) on depression symptoms…”

-----

you didn't give an introductory paragraph for these recommendations. Just added them in the middle without giving context for rationale. It seems like you have learned these recommendations from your primary research questions from your PI's main aims in teh bigger study? Anyways, better to introduce rationale for them and linking them to depressive symptoms reminding the lay reader with that.

The referred sentence is in the center of a paragraph focusing on why the observed factors are important for future digital weight management interventions to consider, and suggesting possible methods that these issues could be accounted for within a future intervention which to my knowledge have not yet been tested in DBCIs. Regarding the insinuation that these suggestions are lifted from “[the] PI’s main aims”, these are my personal, novel contributions into my primary focus of DBCI research that evidence from this study could be used to support. You are welcome to check the cited LNPA primary outcomes paper or clinicaltrials.gov entries to validate this claim, if needed, as considerations for depression are not described in either.

-----

same here, it is better to flush out what they meant by the 2 themes here because they are not understood by the reader - it is not informative at all if the writer assumes the reader should go and read the details of the listed citations in order to understand this paragraph

This is again referring to tailoring as a practice of personalizing intervention content and messages based on participant characteristics. It is very common for tailoring to consider sociodemographic characteristics (e.g., ‘Who I am’), but uncommon to tailor based on psychological characteristics (e.g., ‘How I feel’). This sentence serves to drive home the argument of that paragraph that it is important for DBCI researchers to consider psychological factors such as depression when designing tailored DBCI content.

-----

these constructs are very difficult to separate from the beginning and in this draft you don't seem to have defined them very neatly to assist in measurement. I think you should have constructed the study in a better way statistically and construct wise to avoid this major pitfall.

As stated in the Limitations paragraph, such is the nature of data available for this secondary analysis. My coauthors and I stand by the methodological rigor of this analysis working with the available information from a study which concluded in 2016.

-----

yeah. this is very critical research mistake as it is an essential need for measuring effects and concluding accurate variable relationships. should have been streamlined or cleaned before consducting the analysis because one of the major factors in adherence is the amount of burden it represents to the action taker which is evident in these two examples of self monitoring that you have shared.

As this is an aspect of the parent trial and not this secondary analysis, this is once again a limitation of the data available which is fully acknowledged within this study.

-----

yeah this should have been controlled in study because it is an added burden in addition to self monitoring itself as a behavior

In the Methods section, we note that, “Website page hits refer to the number of time-stamped website URLs visited by logged user IDs. To reduce collinearity with other indicators, the page hits variable only encompasses URLs that did not include the login page, home page, any of the behavioral self-monitoring pages, or the contact/help page. Therefore, increasing values of this variable indicate users accessing more lessons, resources, viewing feedback pages, etc. for a greater breadth of program usage.” This was done to better differentiate measures informing the engagement LV from page hits which would have been necessary to self-monitor, which would be directly related to the adherence LV. It is, however, impossible to do the same for logins, since there is no systematic way to determine when a participant logged into the LNPA website solely to self-monitor for adherence. Moreover, this reinforces the use of SEM to apply ≥3 indicators for each LV, rather than relying on a single indicator of each, and even if such fine-tuning were to have been implemented, it is unlikely that it would meaningfully alter the results or interpretations of this study.

-----

are these technical objective usage metrics "meaningful" for the research questions or clinical outcomes of patients? or they are teh only objective automated data you could get? this is critical to answer for it to be deemed as a strength in your research..

These objective usage metrics are meaningful for this research, as they reflect actual measured performance, and not self-reported participant usage and adherence to program recommendations, which could be influenced by things such as response bias. To address this and other reviewer’s comments, this section has been expanded on to reflect such information.

-----

not meaningful to mention if this was not a charactersitic for this particular study! ...or how did this make this study stronger if I may ask in different way?

On the contrary, the characteristics of the parent study sample can influence the generalizability of findings from a given secondary analysis, which may be informative to varied readership. I have expanded on this statement with the following excerpt:

“Additionally, the parent study included a majority of participants who had not achieved a Bachelor’s degree or equivalent, approximately 25% male participants, included many older adult participants with a wide range of BMIs, and also included many adults living with comorbidities such as diabetes all recruited within primary care settings. Thus, the results and conclusions from this study are likely be somewhat more generalizable to participants recruited for DBCIs within a primary care setting.”
