# Supplementary material for: Measuring the influence of depressive symptoms on engagement, adherence, and weight loss in an eHealth intervention
Source: PLOS Digit Health. 2025 Mar 25;4(3):e0000766. doi: 10.1371/journal.pdig.0000766 (PMC11936179; doi:10.1371/journal.pdig.0000766)
Supplement: Attachment — Submitted filename: Response_to_Reviewers_auresp_2.docx [file pdig.0000766.s004.docx]

Hello all, I organized responses to each item sent from the Editor and Reviewers written in this blue Times New Roman font. Unfortunately, the attached PDF with reviewer comments did not specify the source of each highlighted comment, so I have copy/pasted them with responses in the order received at the bottom of this document. Thank you all for taking the time to review this manuscript.

* A rebuttal letter that responds to each point raised by the editor and reviewer(s). You should upload this letter as a separate file labeled 'Response to Reviewers'. This file does not need to include responses to any formatting updates and technical items listed in the 'Journal Requirements' section below.

* A marked-up copy of your manuscript that highlights changes made to the original version. You should upload this as a separate file labeled 'Revised Manuscript with Track Changes'.

* An unmarked version of your revised paper without tracked changes. You should upload this as a separate file labeled 'Manuscript'.

Reviewers' Comments:

Reviewer's Responses to Questions

Comments to the Author

Reviewer #3: (No Response)

Reviewer #4: I am satisfied with the revisions made.

Reviewer #5: I am reviewing the manuscript “PDIG-D-24-00324_R1_reviewer” where the authors examine how depressive symptoms are associated with engagement and outcome of an eHealth weight-loss intervention.

The study makes a clear contribution to literature. However, I consider the made conceptual and empirical differentiation between “engagement” and “adherence” lacks precision and requires revision.

Major comments:

• As the authors comment, the existing methods for defining and measuring engagement and adherence vary considerably. Here, I find that the two are conceptualized in a way that does not allow differentiating them and contributes to the confusion.

• Conceptually, the authors describe that engagement is “meaningful use”. Adherence is conceptualized as “behavior corresponding with intervention goals”. Are these two definitions not intrinsically related? It appears that adherence is compliance with intervention requests, but these requests should be indications of meaningful use. Thus, if adherence is meeting “meaningful use” goals as defined by researchers, the two concepts are overlapping, which challenges differentiating between them.

As these three comments are closely related, we have attempted to address all three with the reply below the third bullet point, beginning with “Thus,” for conciseness.

• Methodologically, the conceptualization of engagement includes website logins while adherence includes e.g. exercise goals logged. Here, doing the latter requires doing the former, which makes the two concepts naturally linked and correlated.

See below

• Thus, the used conceptual and methodological definitions for “engagement” and “adherence” are interrelated, which confuses the reader. I suggest using either concept, or considerably clarifying on the distinction between “engagement” and “adherence”. This revision should include includes both the “Introduction” section, and “Methods” where the concepts are introduced and then operationalized. Some solutions include conceptualizing engagement as intervention use (omitting the concept of adherence) or showing how engagement is precondition for adherence.

It is true that the two concepts of engagement and adherence are inextricably linked together. The purpose of the included definitions was to operationalize engagement as interacting with the eHealth website, and adherence as their level of actual behavioral performance, which could only be measured by active logging on the same eHealth website. This was an unfortunate aspect of the data available, which is why it is the first limitation described on pg 19., lines 372 -379.

To best address this and reduce collinearity between indicator variables, we distinguished logged user activity to inform different variables based on logged accessing of different webpages (described on pgs 9-10, lines 204-223) as follows:

- Engagement:
  - Logins used website logins
  - Page hits used page URLs *excluding* the login page, home page, any of the self-monitoring pages related to adherence, or the contact/help page.
  - Personal goals set on a supplemental login page (which was not counted towards page hits).
  - Number of social forum posts created, derived from usage data.
- Adherence:
  - Number of weeks logged activity data met minimum program goals, derived from usage data. Multiple days could be entered at once on the website.
  - Number of days participants logged or made any adjustments which would fulfill ≥800 calories consumed as an indicator of complete dietary monitoring, derived from usage data.
  - Number of days participants self-monitored bodyweight. Of all adherence indicator variables, this may be the most correlated with logins if participants only logged in once per day, as the remainder are derived from usage data. While less than ideal, this was necessary to provide a third indicator variable and promote structural equation model identification.

These distinctions are not perfect, but the best that our research team could muster given the nature of data available and have attempted to be as transparent as possible about these limitations. To best address your concerns, we have revised the Introduction sections on pgs 4 and 5 to more clearly indicate how engagement and adherence are known to be conceptually related with one another (excerpts pasted below):

Participant engagement and adherence are both necessary precursors to DBCI effectiveness, which happen more or less in tandem during an intervention. Despite their importance to DBCI research, definitions of these constructs have tended to vary between publications.(8) It is clear that engagement and adherence are inextricably linked process; however, they are conceptually and empirically distinct. Definitions and operationalizations are provided here to promote clarity and comparability.

Engagement is defined here as the extent to which a user interacts with the digital interface of an intervention as intended, based on the systematic definition proposed by Perski et al.(8) Engagement is a latent construct that may be measured using objective usage indicators including logins, page views, proportion of full program components utilized, as well as messages, resources or lessons read.(9, 10)

[…]

Adherence is the next step along the pathway from engagement to successful intervention outcomes, and is defined here as the degree that a participant’s actual (measured) behavior corresponds with requests or goals from an intervention. This definition of adherence is in line with the WHO, and is generally similar to definitions used in other contemporary publications. (16-21)

The authors do not believe further distinctions are necessary within the Methods section, as we have described where and how each indicator was derived, and it would be redundant to restate that logging in was a prerequisite to accessing other parts of the website, as this is mentioned in the Introduction section (pg 7, lines 157-158), and is a commonly understood feature of websites.

• The intercorrelations for Z1-Z7 are now not described. The reader would benefit from such information, which would also rationalize how the constructs that combine these metrics were created. Are the within-group correlations (Z1-Z4 and Z5-Z7) lower/higher/equal than the correlations between groups of Engagement-Adherence?

We have included a CSV of the correlation matrix for indicators Z1-Z7 as a supplemental file in this resubmission and made the following revision to the text:

This high variance in engagement indicators persisted even after outlier testing and removal. Adherence indicators exhibited much lower variability by comparison. A correlation matrix of all indicator variables used in this analysis are available in a supplemental file. Descriptive statistics for all indicator variables are summarized in Table 2.

Minor comments

• “Approximately 53 participants 266 logged in 5 times or fewer over 6 months”, please add percentage after the “53”.

Completed. Excerpt pasted below:

Website engagement indicators exhibited high variability. Approximately 53 participants (14.6%) logged in 5 times or fewer over 6 months, while there were several users with extremely high engagement indicators (e.g., one user had 977 website logins and 4,446 registered page views within 6 months, compared to the sample average of approximately 99 logins and 300 page views).

• “Age was the only consistently significant covariate across both engagement and adherence models, showing positive effects on engagement” in this section, please clarify whether you mean that higher age is associated with higher engagement for instance. Consider clarifying the other statements as well.

These interpretations all follow classic regression model interpretation with a one-unit value increase associated with some change in an outcome controlling for other covariates. We have revised the referred sentence as follows to address this comment:

Age was the only consistently significant covariate across both engagement and adherence models, with increasing participant age above the program mean (51.86) showing positive effects on engagement (γ_eng,age_ = 0.037; SE = 0.008) and adherence (γ_adh,age_ = 0.028; SE = 0.008

• The study should report the nationality of the sample or location of where the sample was drawn from.

Completed. Excerpt pasted below:

Data for this analysis come from LoseNow PA (LNPA), an NIH-funded 12-month cluster-randomized controlled eHealth DBCI for weight management among primary care patients living with overweight or obesity in the United States (clinicaltrials.gov identifier: NCT01606813).(46)

• The reader would benefit from a Figure that illustrates the intervention in the section “Dataset”.

As this is a secondary analysis of the LNPA intervention, we believe this would deviate from the main focus of this study. We frequently cite the primary outcomes article containing full trial details, which interested readers may examine if they are interested.

• I suggest revising the headers. Is “Materials and methods” simply “Methods”? Could the “Dataset” be divided into “Intervention” or “Study procedures” section? Should there be “Participants” section?

The “Materials and Methods” section heading is applied here following the manuscript organization guidelines of this journal. As this is a secondary analysis of a completed intervention, and not reporting on any active outcomes of a study, we would opt to keep the “Dataset” subheading to ensure accuracy and avoid possible reader confusion.

• In Figure 2, are the correlations truly “1” for Engagement-Z1, and Adherence-Z5? If they are, they should be marked with asterisks? Two decimals should be enough precision. Also describe what r stands for.

Figure 2 indicates the factor loadings for each of the indicator variables onto their respective latent variables (LVs). In structural equation modeling, the first indicator for an LV is registered as equal to 1 when examining factor loadings.
